# Coherent Structure of Flow Based on Denoised Signals in T-junction Ducts with Vertical Blades

**DOI:** 10.3390/e21020206

**Published:** 2019-02-21

**Authors:** Jing He, Xiaoyu Wang, Mei Lin

**Affiliations:** School of Energy and Power Engineering, Xi’an Jiaotong University, Xi’an 710049, China

**Keywords:** T-junction, denoise, coherent structure, continuous wavelet transform

## Abstract

The skin friction consumes some of the energy when a train is running, and the coherent structure plays an important role in the skin friction. In this paper, we focus on the coherent structure generated near the vent of a train. The intention is to investigate the effect of the vent on the generation of coherent structures. The ventilation system of a high-speed train is reasonably simplified as a T-junction duct with vertical blades. The velocity signal of the cross duct was measured in three different sections (upstream, mid-center and downstream), and then the coherent structure of the denoised signals was analyzed by continuous wavelet transform (CWT). The analysis indicates that the coherent structure frequencies become abundant and the energy peak decreases with the increase of the velocity ratio. As a result, we conclude that a higher velocity ratio is preferable to reduce the skin friction of the train. Besides, with the increase of velocity ratio, the dimensionless frequency *St* of the high-energy coherent structure does not change obviously and *St* = 3.09 × 10^−4^–4.51 × 10^−4^.

## 1. Introduction

It is well known that the energy consumed by the frictional resistance increases sharply as a train’s speed increases. Due to the high speed of the train, the air passing over its surface is turbulent. The frictional resistance generated by turbulence is much higher than that of laminar flow, which is closely related to the coherent structure [1]. The coherent structure is a structure that is a recognizable orderly large-scale movement. The position and time when it is triggered are not certain, but once triggered, it will develop in a quasi-periodic way and it plays an important role in the transport of mass, momentum, heat and energy [2,3,4,5]. Therefore, it is necessary to study the coherent structures generated by the air passing over the surface of train to lower the frictional resistance. 

The windows of a high-speed train cannot be opened because of the high running velocity, so ventilation openings are an indispensable feature of the train. Their main function is to exchange the inside air with the outside environment, and to import some cold external air for cooling the power equipment. Studying the coherent structure of the flow near the ventilation port could give some guidance for the drag reduction of the train, the selection of materials and the placement of the ventilation ports. In this paper, the flow area near the vent was simplified as the internal flow of the T-junction with a small scale ratio of 1:4.8. The velocity ratio, which is the ratio of the suction velocity of ventilation to the velocity of the train, was proposed to ensure similar flow patterns. More details about the physical model of T-junction have been reported by Su et al. [6].

The T-junction is widely used in the industry field, and many studies have focused on divergent flow and/or combined flow, laminar flow and/or turbulent flow. Beneš et al. [7] dealt with numerical solutions of the laminar and turbulent flows of Newtonian and non-Newtonian fluids in branched channels with two outlets. The results showed that the EARSM turbulence model is capable of capturing secondary flows in rectangular cross-section channel. Neofytou et al. [8] performed numerical investigations on the shear-thinning and shear-thickening effects of flow in a T-junction of rectangular ducts. The results demonstrated the extent of the effect of the *Re* number on the velocity profiles at different positions in the domain for both Newtonian and non-Newtonian cases. Wu et al. [9] investigated the breakup dynamics of ferrofluid droplets under magnetic fields in a microfluidic T-junction. Chen et al. [10] computed a global linear sensitivity analysis of a complex flow through a pipe T-junction. They found that when *Re* ≥ 320, the T-junction contains four instances of a bubble-type vortex-breakdown-like flow feature, where the dynamics are highly sensitive to spatially localized feedback, especially near the boundaries of the recirculation regions. Lu et al. [11] discussed how a T-junction, as a separator in an emerging thermodynamic cycle, affects the cycle efficiency.

Wavelet analysis is a powerful tool for studying turbulence characteristics. The continuous wavelet transform (CWT) has been used by many scholars to analyze the coherent structures in flows. Baars et al. [12] studied the modulation mechanism of small scale flows by large-scale motions, and the results revealed that the time shift in frequency modulation is smaller than that in amplitude modulation. Besides, the wavelet Morlet was used in their study to analyze turbulent signals. Bulusu et al. [13] detected the coherent structure of a curved artery model by means of CWT and decomposition (or Shannon) entropy. They concluded that the optimal wavelet-scale search was driven by a decomposition entropy-based algorithmic approach and proposed a threshold-free coherent structure detection method. The method was successfully utilized in the detection of secondary flow structures in three clinically-relevant blood flow scenarios. CWT was used to detect and establish the length and time scales of the largest horizontal coherent structures existing in a shallow open channel flow by Kanani et al. [14]. They found CWT was particularly well suited to determine the average time and length scales of the structures. Individual horizontal coherent structures whose characteristic times approximately twice larger than the value of average time scale could be identified with the method. Both the Reynolds and wavelet methods were utilized to analyze a solitary wave, a gravity wave, a density current and a low-level jet in the stable atmospheric boundary layer by Ferreres et al. [15]. The results indicated that wavelet analysis had the capacity to distinguish the different scales involved in all these events. Wang et al. [16] investigated the multi-resolution characteristics of velocity components using Morelet and db3 wavelets. It was found that the energy cascade of the drag reducing flow was greatly suppressed with fewer energy branching with the existence of polymer additives. Sarma et al. [17] discussed the self-similarity properties of turbulence in magnetized DC glow discharge plasmas by evaluating the Hurst exponent from wavelet variance plots. The exact frequency responsible for the chaotic behavior could be further determined. In addition, the Morlet wavelet is widely used to analyze turbulent or velocity signals [14,18,19], which indicates that the Morlet wavelet could be one of the options for the CWT in our study.

Several papers about simplified T-junction models of the vents of high-speed trains have been published by the members of our group. They mainly focused on the distribution and high-order statistics of the velocity in cross ducts, as well as the distribution of pressure in branch ducts [20,21,22,23]. Atzori et al. [24] studied the coherent structure of a square duct, and the results showed that the large-scale structure attached to a horizontal wall has the same size as that of a vertical wall. As the Reynolds number increases, the size of the coherent structure is bigger. Su et al. [6] studied multi-resolution coherent structures in T-junctions without blades, but they did not eliminate the edge-effect of the CWT. In fact, there are typically blades in the vents of the train to prevent waste from entering the train cabin. In this study, four blades were set at the entrance of the branch duct, and denoising was performed. Besides, the edge-effect that may lead to a wrong result in CWT was eliminated. Thus the results were closer to a real situation. This is beneficial for controlling the flow resistance generated by high-speed train vents and ultimately improving the train efficiency.

## 2. Experimental Setup

**Figure 1:**
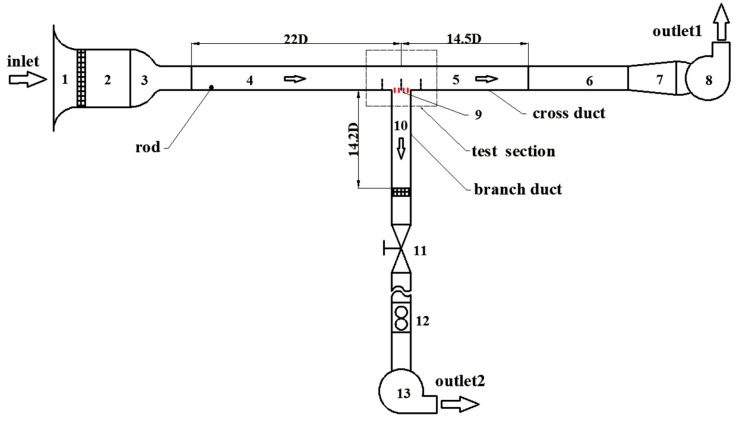
Schematic diagram of experimental system: 1-Entrance, 2-Settling chamber, 3-Contraction section, 4-Front section of cross duct, 5-Back section of cross duct, 6-Connect section, 7-Expansion section, 8-Fan, 9-Blades, 10-Branch duct, 11-Valve, 12- Glass rotameter, 13-Fan.

**Figure 2:**
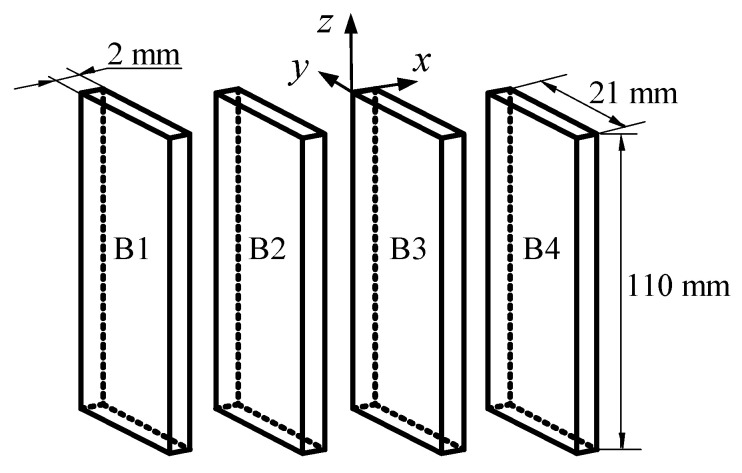
Schematic diagram of the blades.

**Figure 3:**
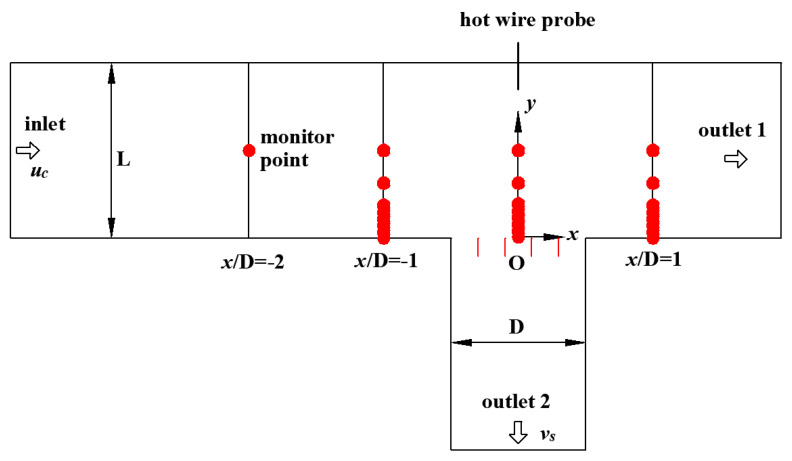
Sketch of the measurement points.

To investigate the influencing factors of the flow near the vents of high-speed trains, an experimental system in the form of T-junction was built to mimic a real situation. This experiment was carried out in a low-speed wind tunnel with a maximum speed of 57 m/s. A schematic diagram of experimental system is illustrated in Figure 1. The cross duct has a length of 36.5 D and its cross-sectional area is *z* × *y* = 161.7 × 143.3 mm^2^. The branch duct is 14.2 D long and its cross-sectional area is a square with a side length of D = 110 mm. To simulate the grating of the train vent, four vertical blades were mounted equidistantly on the inlet section of the branch duct. Each of them is 2.0 × 21.0 × 110.0 mm^3^ in size, as shown in Figure 2. In order to ensure that the turbulence is fully developed as soon as possible, a 2.0 mm rod was placed in the entrance of cross duct. The coordinate system is used herein, the origin of the coordinate is at the inlet center of the branch duct (as shown in Figure 3). The *x*, *y* and *z* axes are aligned with the streamwise, wall-normal and spanwise directions of the cross duct, respectively.

In this experiment, the flows at different positions and different cross sections were analyzed by varying the velocity of the cross duct and the velocity ratio *R* (the ratio of the branch velocity to the cross velocity). The cross velocity was measured by an IFA300 hot wire anemometer and regulated by changing the cross flow using the high-power inverter fan. The sampling frequency was 50 kHz. The sampling time was 40.6 s, and the uncertainty of the cross velocity was ±1.0% after calibration. When the cross velocity was constant, the velocity ratio was varying with the velocity of the branch duct which was controlled by a low-power inverter fan. The flow rate of the branch duct was measured by a glass rotameter, and the uncertainty of the bulk branch velocity was ±4.3%. More details on the experimental setup have been published in Yin et al. [20] and Su et al. [6].

The centre velocities of the cross duct in the experiment were 30, 40, and 50 m/s, respectively, and the velocity ratios were 0.08, 0.13, and 0.18. The experimental conditions are listed in Table 1. The measurement sections were *x*/D = −1, *x*/D = 0, *x*/D = 1 and eight measurement points were selected varying from *y*/L = 0.0070 to 0.5000 at each section as presented in Figure 3. Note, L is the height of the cross duct. All processing and analysis of data were carried out with the MATLAB software.

In order to ensure the reliability of the experimental system, the velocity distribution curve obtained at *x*/D = −3 was compared with the results obtained by Gessner et al. [25].

**Figure 4:**
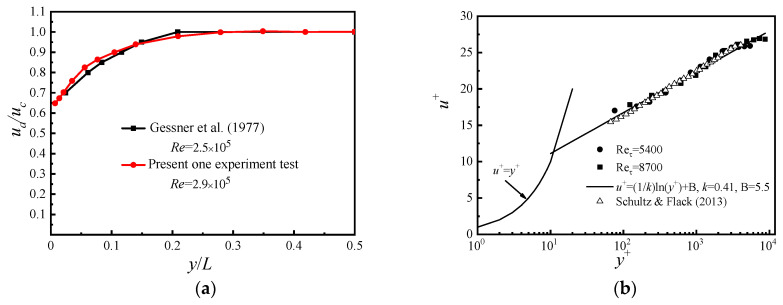
(**a**) Comparison of velocity distribution between our experiment and Gessner’s et al.; (**b**) Comparison of wall velocity distribution between our experimental data and results of Schultz and Flack.

The results are shown in Figure 4a and consistency between our experiment and Gessner’s was found. The wall velocity distribution curve at *x*/D = −5 was compared with the results of Schultz and Flack [26], and the result is shown in Figure 4b. There is also a good conformity. Detailed information can be found in Su et al. [6].

The accuracy of the hot-wire anemometry suffers from several problems, such as the heat conduction to the wall, calibration at low velocities, spatial resolution (due to the wire length *l*) and determination of the wall position (due to heat conduction) and the risk of probe damage. The error due to the spatial resolution is maximized at only 13% at *y*/L = 0.0070, then sharply reduces to 6% at *y*/L = 0.014. Therefore, it could be thought that the error is systematic due to the same hot-wire probe, and this does not adversely affect the results near the T-junction [6]. The inaccuracy because of the blockage effects can be negligible for $y^{+}$ values larger than 20 wall units, and the minimum $y^{+}$ in our study is 76, so the effect of blockage can be negligible [27].

## 3. Processing of Data

In this paper, the coherent structures were mainly analyzed by performing CWT on the obtained signals. CWT means that a convolution was executed on the obtained signals and the selected wavelet basis function where the scale dilation *a* is continuous. Its expression is shown in Equation (1):

(1) $$W_{f}\left(a,b\right)=\overset{+\infty }{\underset{-\infty }{\int }}f\left(t\right)\psi_{a,b}\left(t\right)dt=\langle f\left(t\right),\psi_{a,b}\left(t\right)\rangle$$

where:

(2) $$\left\{\psi_{a,b}\left(t\right)=\frac{1}{\sqrt{a}}\psi \left(\frac{t-b}{a}\right)|a>0,b\in R\right\}$$

The variables *a* and *b* in these equations are commonly called the scale dilation and translation parameters, respectively [28]. f(t) is the obtained signal. The translation parameter, *b*, corresponds to the position of the wavelet basis function in the analyzed signal in time, and the scale dilation parameter, *a*, reflects the degree of dilation of the wavelet. The *a* has the following correspondence with frequency:

(3) $$f=f_{c}\times \frac{f_{s}}{a}$$

where *f* is the corresponding scale frequency, which reflects the frequency (scale) of the signal at the certain moment and scale dilation a. *f*_c_ is the center frequency of wavelet basis function, and *f*_s_ is the sampling frequency.

To analyze coherent structure, the CWT power spectrum of a signal is necessary. It can be defined as follows [14,18,29]:

(4) $$E_{c}(a,b)=\int_{-\infty }^{\infty }{\left|W_{f}(a,b)\right|}^{2}$$

### 3.1. Denoising

During the acquisition of turbulent signals, the obtained signals are actually the mixture of real signals and noise signals due to the influence of measuring devices and ambient noises. The obtained signals can be expressed as the following form: *o*(*t*) = *s*(*t*) + *n*(*t*), where the *o*(*t*) is the obtained signal, the term *s*(*t*) is the ideal real signal and the *n*(*t*) is the noise signal caused by the measurement process. When CWT is performed on the obtained signal to analyze coherent structures, the presence of noise may affect the results, so denoising is a necessity. The aim is to remove the noise signal *n*(*t*) and obtain the ideal real signal to the utmost extent.

Many denoise methods can be applied, such as Gaussian filters, wavelet filters and deep learning. The Gaussian filter method is more suitable for stationary signals, and deep learning is suitable for images. The wavelet method can decompose signals into the frequency domain and the time domain, and will not smooth out the instantaneous component of signals during the decomposition and reconstruction process. Turbulent signals have the characteristics of being instantaneous and pulsating. It is important to maintain the spikes and abrupt changes during the denoising process, so the wavelet filter is a powerful tool for denoising turbulent signals. Common wavelet denoising methods are based on modulus maxima, translation invariant, and threshold. Among them, the method based on threshold can fully consider the propagation characteristics of signals and noises at different scales. Meanwhile it is simple to calculate and distinguish the signals from the noises. Therefore, the wavelet threshold method was used in this paper to denoise the signals.

In the actual processing, it is widely believed that the noises are distributed in the high frequency band. Thus, only the high frequency coefficients need to be processed. The parameters that need to be considered for wavelet threshold denoising include the threshold function, threshold, decomposition layer number and wavelet basis function. The process is described as follows:

Step (1): Wavelet decomposition of signals. Select the basis function and the number of decomposition layers to perform wavelet decomposition on signals;
Step (2): Perform threshold denoising on the high frequency coefficients. The high frequency coefficients of each decomposition layer are processed using the selected threshold and threshold function;
Step (3): Signal reconstruction. Wavelet reconstruction is performed with the processed high frequency coefficient and the low frequency coefficient of the largest decomposition layer.

The noises are often regarded as white noises, that is, obeying a Gaussian distribution. Corresponding to the process of denoising using the method of wavelet threshold, that is, the energy of real signals can be centralized on a few wavelet coefficients, while the energy of noises is distributed on most wavelet coefficients. By choosing an appropriate threshold, most of the noises can be removed and the best approximation of the ideal real signals can be obtained. Commonly used threshold functions are the hard threshold and soft threshold function. In a hard threshold function, the coefficients with absolute values lower than the threshold are set to zero; in soft one, beyond that, the coefficients with absolute values higher than the threshold are shrunk. The hard threshold function will be discontinuous at the threshold, which will bring about the Gibbs phenomenon, and furthermore distorts the reconstruction. Thus there may be false coherent structures because of the distortion. The soft threshold function has a constant error due to shrinking and the reconstruction result is not accurate. Thus the energy value of coherent structure may be decreased and the boundary of the coherent structure is not clear. Therefore an improved threshold function is necessary. It should provide a smooth transition at the threshold position and should be extremely approximate away from the threshold. Many improved functions have been proposed [29,30,31,32]. The sigmoid function proposed by Yi et al. [33] was chosen in this paper, and its expression is described as follows:

(5) $$\bar{d_{i,j}}=\{\begin{array}{ccc} (\left|d_{i,j}\right|-t)-\left[\frac{2}{1+e^{{}_{\beta (\frac{\left|d_{i.j}\right|-t}{t})}}}\right] & , & \left|d_{i,j}\right|\geq t\\ 0 & , & \left|d_{i,j}\right|<t \end{array}$$

where: β = 10, the *d_i,j_* is the coefficient obtained by CWT, and the *t* is the value of threshold. This function can overcome the shortcomings of the hard threshold and soft threshold functions to some extent, and offers better denoising performance.

The threshold is also one of the important parameters in the denoising process. An excessive threshold will eliminate useful components in the signals, and a smaller threshold will lead to insufficient denoising. The commonly used threshold $t=\sigma \sqrt{2\ln N}$ was proposed by Donoho [34]. Here *σ* is the standard deviation of the noise and *N* is the length of the signal. The aforesaid threshold is a fixed threshold, and the same value is used in different decomposition layers, which may erase some useful signals. In this paper, the threshold proposed by Lu [30] was chosen:$t=\sigma \sqrt{2\ln N}/\log2(j+1)$, where *j* is the layers in the decomposition. The thresholds vary with the decomposition layers, and are consistent with the propagation characteristics of noise.

An appropriate basis function is the key point of obtaining the desired features of signals. An ideal basis function should satisfy the following characteristics: biorthogonality, compact support, regularity and symmetry. Combining the above characteristics and the results of Zhang [35], the sym5 wavelet was used as basis function in denoising for this article. In the actual processing, the noises are often regarded as white noises, that is, obeying the Gaussian distribution, so the value of the decomposition layer is often chosen based on the theory of verification of white noise That is, if the wavelet coefficients at a certain decomposition layer are verified to satisfy the characteristics of white noise, the current layer is the goal decomposition layer-number. The “white noise test” proposed by Zhang et al. [36] was used for reference in denoising for this study, but errors may occur when the sample size is small, so an improved method based on K-S test was used and finally two layers were chosen for denoising.

### 3.2. Eliminating of Edge Effect

When executing CWT to analyze signals, the ideal length of the signal is infinite, but the measured signal has a finite length, so errors occur at the start and the end of the transformed signal. These errors are called the edge effect or the cone of influence (COI) [37,38,39]. The edge effect causes false peaks at the edges, affecting the acquisition of correct results, therefore, it is necessary to eliminate the edge effect. The radius of COI depends on the selected wavelet basis function and scale. As the scale increases, the area affected by COI also increases.

For CWT analysis of turbulence, the most common wavelet basis function is the Morlet wavelet [7,14,18]. It is a complex-valued wavelet and can provide not only phase information, but also amplitude information. Furthermore, it has a good local balance in both the time and frequency domains [6], so the Morlet wavelet was used in the processing of CWT in this study. Its definition is:

(6) $$\varphi (t)=\pi^{-1/4}e^{i\omega_{0}t}e^{-t^{2}/2}$$

where *ω_0_* is the dimensionless frequency and here taken to be *ω_0_* = 6 following the recommendations of Torrence et al. [37].

According to the definition of Torrence [37], the radius of COI of Morlet wavelet is the e-folding time (*e*^−2^) of the wavelet power autocorrelation at each scale. That means the wavelet power for a discontinuity at the edge drops by a factor of *e*^−2^ and this ensures that the edge effects are negligible beyond this point. In addition, referring to the definitions of Boltezar et al. [38] and Mayer et al. [39], the radius of COI was finally selected as 2*a* (*a* is the scale of CWT).

The steps for eliminating the edge effects are described as follows:

Step (1): Extension. Extend the signal to be analyzed by the abovementioned length 2*a*;
Step (2): CWT transform. Perform CWT on extended signals obtained after Step (1);
Step (3): Truncation. Truncate the transformed signal after Step (2) to the same length as the original signal.

## 4. Results and Discussion

**Figure 5:**
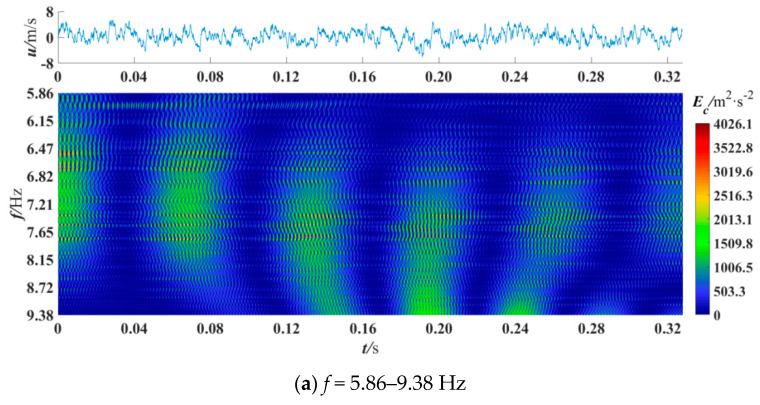

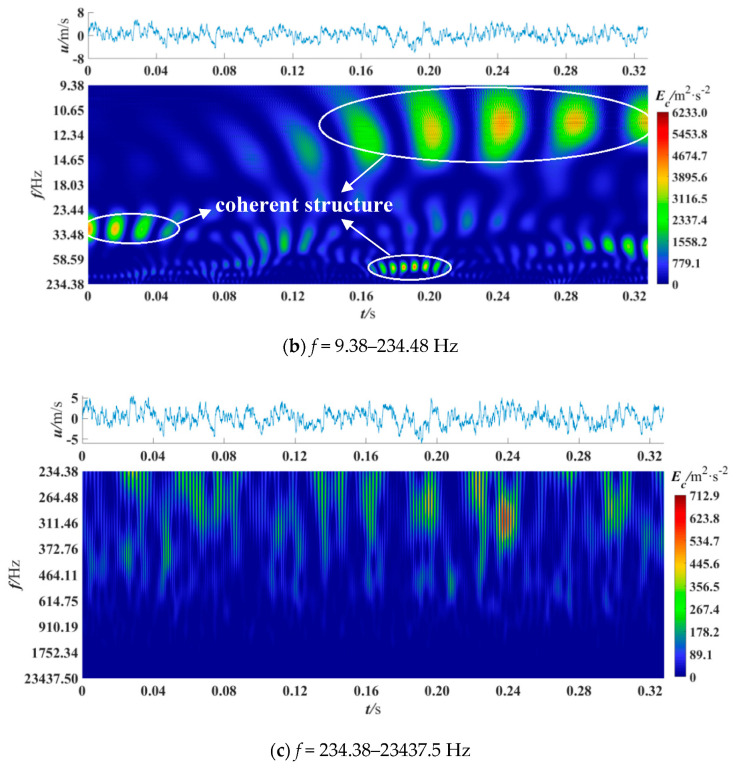
Wavelet power spectrum at *x*/D = 0, *R* = 0.13, *u*_c_ = 40 m/s, *y*/L = 0.0070.

**Figure 6:**
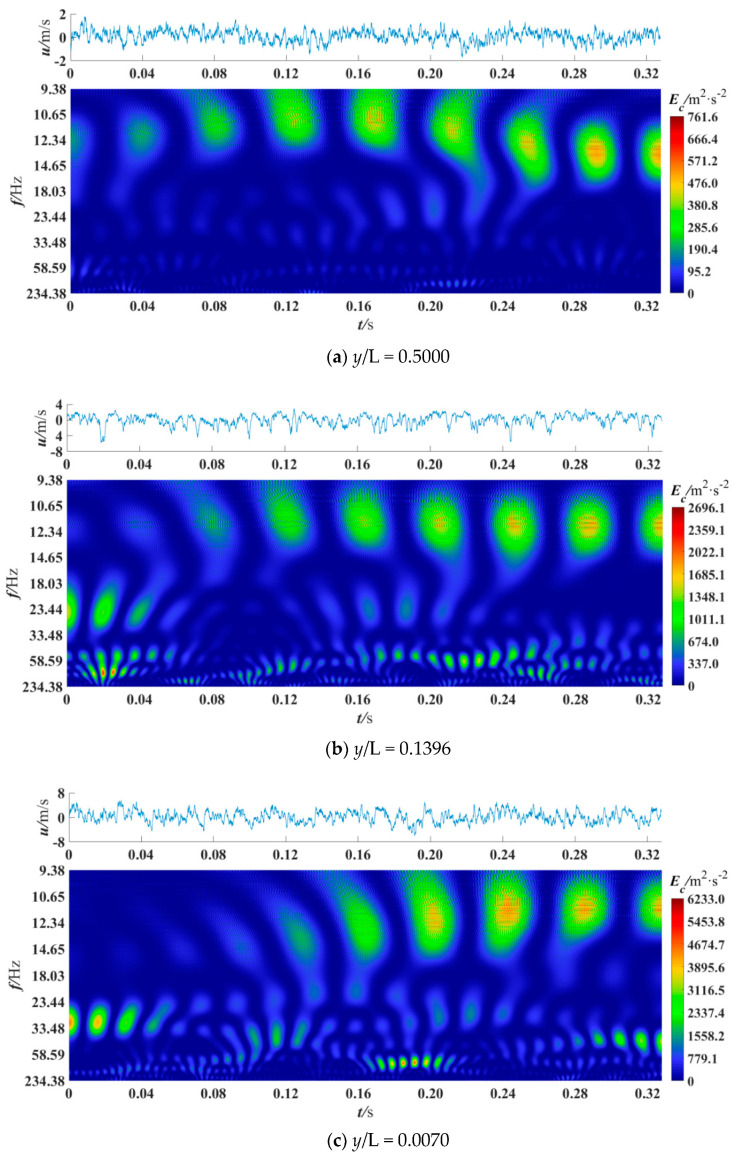
Wavelet power spectrum at *x*/D = 0, *R* = 0.13, *u*_c_ = 40 m/s.

**Figure 7:**
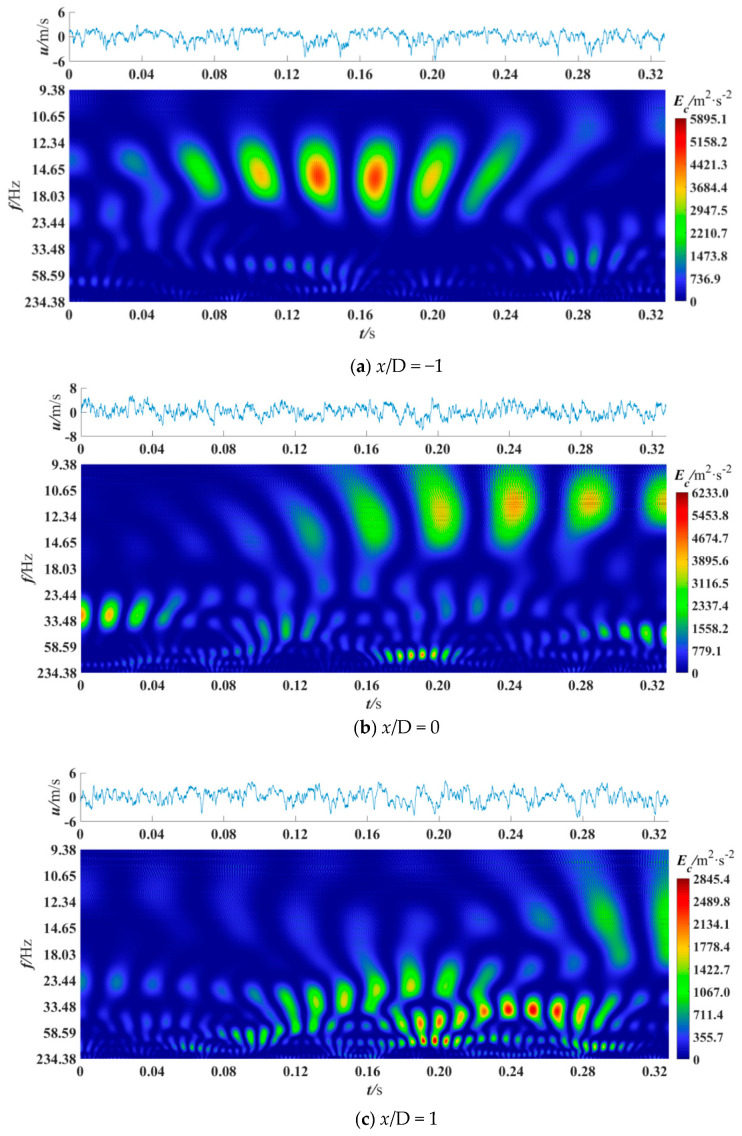
Wavelet power spectrum at *u*_c_ = 40 m/s, *R* = 0.13, *y*/L = 0.0070.

**Figure 8:**
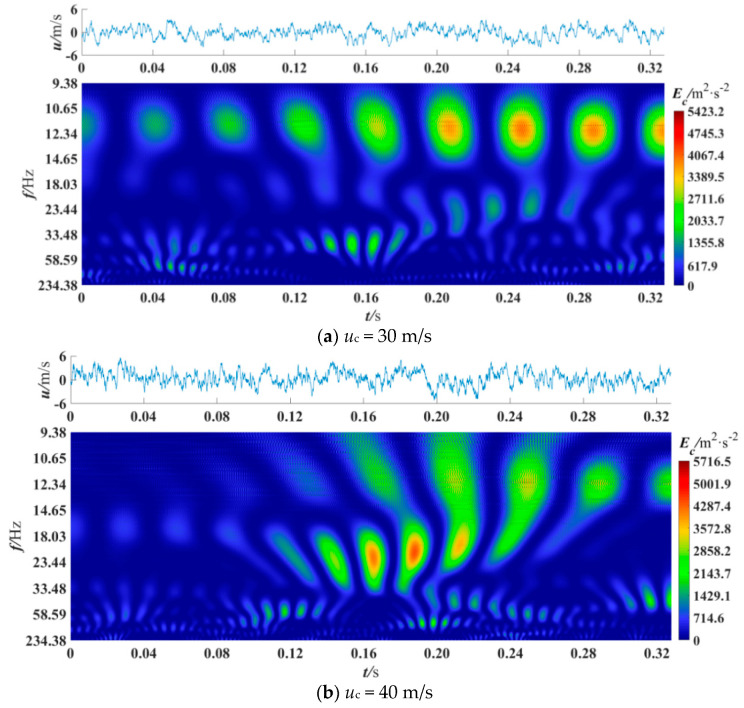

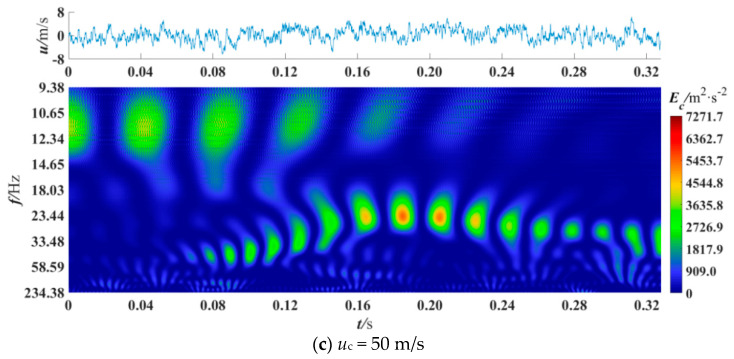
Wavelet power spectrum at *x*/D = 0, R = 0.13, *y*/L = 0.0070.

**Figure 9:**
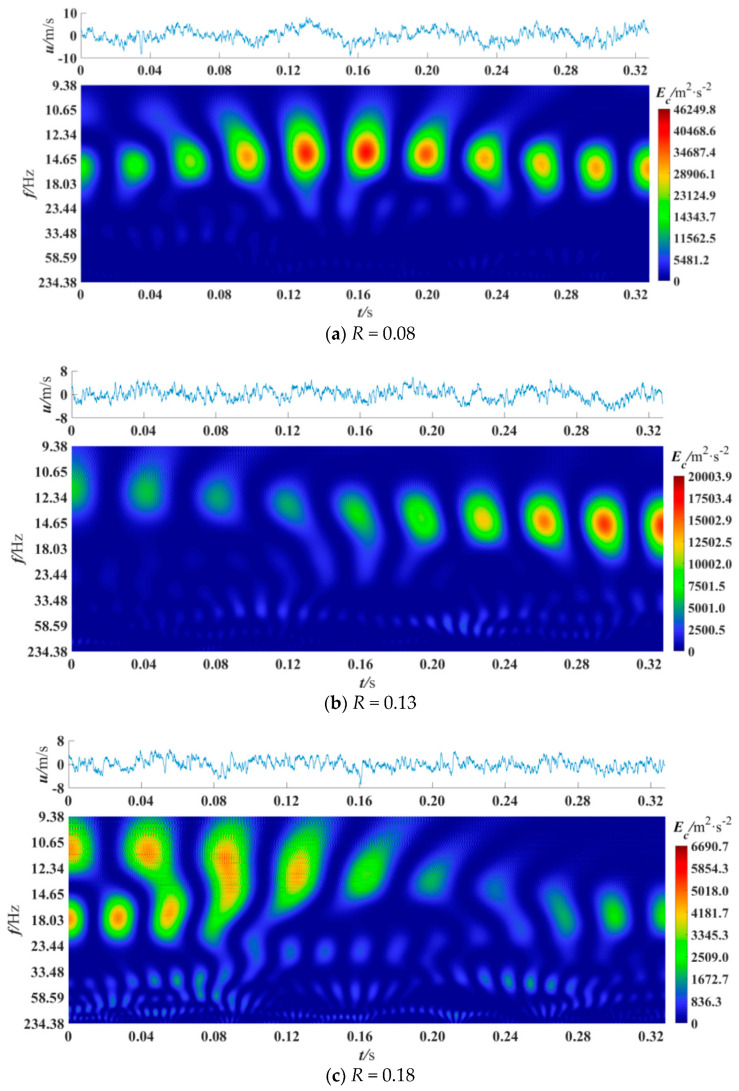
Wavelet power spectrum at *x*/D = 0, *u*_c_ = 40m/s, *y*/L = 0.0070.

The wavelet coefficients obtained by CWT represent the information of coherent structures with different scales in turbulence [18], and the power spectrum represents the energy of coherent structures with different scales [14]. In this section, CWT was used to obtain the power spectrum of the denoised fluctuating velocity signals to analyze the coherent structure. Besides, the edge effect generated on CWT was eliminated. The cross velocity and velocity ratio were varied to observe the power spectrum differences in different sections and positions. A total of 16384 data points were selected for analysis, and previous studies have shown that this amount of data is sufficient [7,40]. Figure 5, Figure 6, Figure 7, Figure 8 and Figure 9 are the wavelet power spectra of the fluctuating velocity under different parameters, which mirror the time-frequency characteristics of the coherent structures. In all following figures, the abscissa refers to time, and the ordinate refers to frequency transformed from scale dilation *a*. The colorbar on the right expresses the wavelet power defined in Equation (4). The wider the spectrogram band along the abscissa is, the larger the scale of the coherent structure is.

The Strouhal number is often used to give a non-dimensional description of the flow characteristics of a periodic flow [41,42]. Coherent structures have quasi-periodic characteristics, so the Strouhal number (*St*) is employed to describe the frequency characteristics of coherent structures. The *St* is defined as follows:

(7) $$St=\frac{yf}{u_{c}}$$

where *y* is the distance from the wall, and the *f* is the main frequency component of the coherent structure. Figure 5 shows the power spectrum under different frequency ranges at the first measured point, *y*/L = 0.0070, where the minimum value is 5.86 Hz and the maximum is 2,3437.50 Hz and other parameters are *u_c_* = 40 m/s, *R* = 0.13, *x*/D = 0. 

In Figure 5a, large-scale high-energy coherent structures can be found over the whole time period with a scale frequency range of 5.86–9.38 Hz, but they are not very clear. The high-energy coherent structure was defined as those whose value of energy accounts for more than 33% of the maximum value, as shown in Figure 5b. There, except for the five large-scale coherent structures with the dominant scale frequency of 12.34 Hz, there are three coherent structures at 0–0.04 s with a dominant scale frequency of 30 Hz. Coherent structures with a scale frequency of 60 Hz can be found at 0.10–0.12 s and 0.29–0.32 s. There are also coherent structures at 0.17–0.20 s whose scale frequency is more than 100 Hz. The presence of large-scale coherent structures is hardly observed in Figure 5c. What’s more, the peak value of energy is *E*_c_ = 712.9 m^2^/s^2^, or 7.7 times lower than that in Figure 5b. The above description shows that coherent structures with large energy exist with a frequency of 9.38–234.38 Hz, and they play a vital role in the transport of mass and energy. Therefore, the frequency of the coherent structures studied in this paper ranges from 9.38–234.38 Hz (corresponding to scale dilation *a* of 200–5000), which is consistent with the results of Su et al. [6]. This may indicate that the presence of blades does not remarkably change the frequency range of coherent structures.

Figure 6 shows the wavelet power spectrum at different locations away from the wall (*y*/L = 0.0070–0.5000). They were all measured with *x*/D = 0, *R* = 0.13, *u_c_* = 40 m/s. In Figure 6a, at *y*/L = 0.5000, the coherent structures with the maximum energy value of *E*_c_ = 761.6 m^2^/s^2^ appear at 0.08–0.32 s, whose dominant scale frequency is 9.38–14.65 Hz. In Figure 6b, at *y*/L = 0.1396, besides the coherent structures of 12.34 Hz at 0.12–0.32s, the coherent structure of 23.44 Hz appears in 0–0.04 s. The coherent structures with the largest energy value appear in the high-frequency band of about 100 Hz. The peak value of energy is 2696.1 m^2^/s^2^. In Figure 6c, at *y*/L = 0.0070, the scale frequency of the coherent structures is wider, including low-frequency of 9.38–14.65 Hz, 24–40 Hz and some high-frequency coherent structures of 100 Hz and greater than 100 Hz. The peak value of energy is 6233.0 m^2^/s^2^, which is 7.2 times higher than that in Figure 6a and 1.3 times higher than that in Figure 6b. The similarity among the three figures is the periodic distribution of coherent structures: they are all periodic in term of time but in an uncertain period. That is consistent with the characteristic of quasi-periodic of the coherent structures. And the tendency of large-scale coherent structures separating into small-scale coherent structures can also be seen. That means the event of energy cascade occurring. However, in areas far from the wall, the coherent structure contains less energy and the frequency range is not as big as the near wall area. That indicates that there are more coherent structures triggered near the wall (*y*/L = 0.0070). Therefore, in the following figures, the distribution of coherent structures in the near-wall region was mainly concerned.

Figure 7 presents wavelet power spectra at different cross sections. The other parameters are *u_c_* = 40 m/s, *R* = 0.13, *y*/L = 0.0070. In Figure 7a, at *x*/D = −1, there are six large-scale high-energy coherent structures with a frequency of 12.34–18.03 Hz at 0.08–0.24 s, and the peak value of the energy is 5895.1 m^2^/s^2^. In Figure 7b, at *x*/D = 0, the coherent structures span a wider scale frequency range. Within 0.16–0.32 s, there are six high-energy coherent structures with the scale frequency about 12.34 Hz. There are four coherent structures with a frequency of 30 Hz at 0–0.04 s, and six small-scale high-frequency (more than 100 Hz) coherent structures at 0.17–0.20 s. Coherent structures with a scale frequency of about 60 Hz also exist at 0.10–0.12 s and 0.29–0.32 s, respectively. The peak value of energy is 6233.0.4 m^2^/s^2^. 

In Figure 7c, at *x*/D = 1, the coherent structures are mainly concentrated in the middle-frequency and high-frequency above 23.44 Hz at 0.12–0.28 s, and coherent structures with a wide scale frequency range (33.48–234.38 Hz) are periodically passed. At 0.30 s and 0.32 s, two coherent structures with the abundant scale frequency components of 12.34–18.03 Hz can be seen if looking carefully. In summary, upstream, the coherent structures are mainly low-frequency large-scale structures, and *St* = 3.09 × 10^−4^–4.51 × 10^−4^, which indicates that without the influence of blades. At the mid-center and downstream, the frequency components of the coherent structures are richer, and *St* = 3.09 × 10^−4^, 7.50 × 10^−4^, 15.00 × 10^−4^ and 25.00 × 10^−4^. That may because the suction of the branch duct accelerates the energy cascade. Therefore, with the existence of the branch duct suction, the *St* in a square section is mainly 3.09 × 10^−4^ –25.00 × 10^−4^. To reduce the flow resistance, the formation of coherent structures with the corresponding scale frequency at the upstream and mid-center locations should be suppressed.

Figure 8 illustrates the wavelet power spectrum at the near wall point under different cross velocity conditions and a given velocity ratio (*R* = 0.13). It can be seen from Figure 8a that, at *u_c_* = 30 m/s (*Re*_c_ = *u*_c_⋅ L/ν = 2.85 × 10^5^, ν is the kinematic viscosity), there are six clear large-scale coherent structures periodically passed through at 0.12–0.32 s, and their scale frequency range is 10.65–14.65 Hz. There are four small-scale coherent structures at 0.14–0.16 s and their scale frequency range is 33.48–58.59 Hz. The coherent structure with the highest energy appears at 0.24 s with an energy peak value of 5423.2 m^2^/s^2^. In Figure 8b, at *u_c_* = 40 m/s (*Re_c_* = 3.8 × 10^5^) five large-scale coherent structures with high-energy are present at 0.18–0.32 s. The phenomenon can be seen at 0.20–0.24 s that the low-frequency coherent structures tends to break into higher-frequency ones. At 0.12–0.24 s, coherent structures with a scale frequency band of 18.03–33.48 Hz are periodically passed through. The coherent structures with highest energy are present at 0.16–0.18 s and their energy value is 5716.5 m^2^/s^2^. 

In Figure 8c, at *u_c_* = 50 m/s (*Re_c_* = 4.75 × 10^5^) there are four large-scale coherent structures at 0–0.12 s with a dominant scale frequency of 11 Hz, and the highest energy coherent structures appear at 0.16–0.20 s with the scale frequency band of 23.44–33.48 Hz. A number of small-scale coherent structures with frequency of 50 Hz and 33 Hz are seen at 0.08–0.32 s. As the cross velocity increases, the peak value of energy also increases.

With the increase of *Re_c_*, the frequency band of the coherent structures is wider, which are *St* = 3.55 × 10^−4^–4.88 × 10^−4^ for *Re_c_* = 2.85 × 10^5^, *St* = 2.66 × 10^−4^–8.37 × 10^−4^ for *Re_c_* = 3.8 × 10^5^ and *St* = 2.20 × 10^−4^–10.00 × 10^−4^ for *Re_c_* = 4.75 × 10^5^. Therefore, it could be concluded that if the speed of train is increased without changing the vent speed, the energy consumed by the friction near the ventilation would also be increased. Comparing to the results in Ref. [6], the periodicity of coherent structure is more obvious and the value of energy is lower. This may indicate that the existence of blades could rectify the flow and lower the intensity of the turbulence. Thus we recommend that an appropriate number of blades could be helpful to reduce the skin friction of trains. The appropriate number needs to be studied further.

Figure 9 presents the wavelet power spectrum at a given cross velocity *u*_c_ = 40 m/s (*Re_c_* = 3.8 × 10^5^) under different velocity ratios. From Figure 9a, at low velocity ratio *R* = 0.08, it is demonstrated that the coherent structures periodically pass through over the whole time period, and their dominant scale frequency is 14.65 Hz. The coherent structures with highest value of energy appear from 0.13 to 0.16 s, and the maximum value is 46249.8 m^2^/s^2^. In Figure 9b, at *R* = 0.13, coherent structures with lower energy are spread from 0 to 0.16 s, and their dominant scale frequency is gradually increased. In addition, high-energy coherent structures periodically exist at 0.16–0.32 s, and their dominant scale frequency is 14.65 Hz. In Figure 9c, at high velocity ratio, *R* = 0.18, there are coherent structures periodically passing through at the time period of 0–0.16 s, and the dominant scale frequencies of them are 11 Hz for large-scale and 18.03 Hz for mid-scale respectively. Coherent structures spanned the frequency band of 9.38–23.44 Hz can be clearly seen at 0.05–0.08 s. This indicates that there is a tendency for large-scale coherent structures to break into higher frequency and smaller scale ones, which is called an energy cascade. Viewing the above three figures, it can be concluded that as the velocity ratio increases, the energy peak of the coherent structures decreases, and the energy cascade phenomenon becomes obvious. This may be because a smaller velocity ratio means a higher velocity in the cross duct when the cross velocity is the same, and the higher velocity could thin the laminar sub-layer near the entrance of the branch duct and motivate more violent turbulence, which in turn generates more coherent structures. Moreover, when the velocity ratio is smaller, the variance of the pulsation velocity is larger. At *R* = 0.08, the variance of the pulsation velocity is 7.31m^2^/s^2^, 3.86 m^2^/s^2^ at *R* = 0.13, and 3.18 m^2^/s^2^ at *R* = 0.18. That means bursting events could increase under a lower velocity ratio. Therefore, increasing the velocity ratio may be advantageous to accelerate the energy cascade and suppress the generation of high-energy coherent structures. The acceleration and suppression suggest that the drag could be reduced by increasing the velocity ratio of the train. Besides, the local *St* doesn’t change with the increase of the velocity ratio. The *St* of the coherent structure that contains high energy and with a large scale is 3.09 × 10^−4^–4.51 × 10^−4^.

From Figure 5, Figure 6, Figure 7, Figure 8 and Figure 9, we can summarize that, at the position of *y*/L = 0.0070 and *x*/D = 0, the main frequency of coherent structure is *St* = 2.20 × 10^−4^–10.00 × 10^−4^. There more large-scale coherent structures triggered near the wall (*y*/L = 0.0070), which means an intense turbulent event, and the quasi-periodic characteristics of the coherent structures have no relation with the intensity of the turbulent event. Because of the suction of the branch duct, the scales of coherent structures become more abundant, and the maximum frequency reaches *St* = 25.00 × 10^−4^. This means the existence of the suction accelerates the mass and energy transport process. The higher cross velocity is helpful to the generation of coherent structures and the blades at the entrance of branch duct could weaken the process of coherent structure triggering. The higher velocity ratio would result in lower energy coherent structure peaks.

There is a secondary flow because of the square shape of the duct which is related to the secondary shear Reynolds stress and centrifugal force. The interaction of the motion of sweep and injection generated from the side wall influences the wall-shear stress and heat transfer performance. The velocity fluctuation in the outer region is stronger than that in the circular pipe [43], so the coherent structures in rectangular ducts may be different from those in circular pipes. Accurate coherent structure results should be studied further considering the influence of secondary flow.

## 5. Conclusions

In this paper, the vent of a high-speed train is simplified as a T-junction duct with vertical blades. The velocities at three different locations, i.e., upstream, mid-center and downstream, were measured by a hot wire anemometer. The velocity signals were denoised with the wavelet threshold denoising method, wherein the threshold function is improved. The wavelet power spectrum was obtained by CWT, and the coherent structures in the T-junction under different conditions were analyzed, while the COI was eliminated. The following three main conclusions are drawn:

1. The coherent structures in the upstream region of a T-junction are mainly low-frequency. There are more abundant frequency components of coherent structures at the mid-center and downstream. The *St* of the coherent structure in this study is ranging from 3.09 × 10^−4^ to 25.00 × 10^−4^. The energy of coherent structures is the highest at the mid-center, and suppressing the formation of low-frequency coherent structures at the upstream and mid-center may be beneficial to reduce the drag force.
2. With the increase of *Re_c_*, the energy peak of the coherent structures also increases and the frequency range of coherent structures is more abundant. The dimensionless frequency *St* changes from 3.55 × 10^−4^–4.88 × 10^−4^ to 2.20 × 10^−4^–10.00 × 10^−4^. Therefore, the energy consumed by friction may be increased with the improvement of speed of train. The existence of blades is helpful to reduce the skin friction of the train.
3. When the velocity ratio increases, the energy peak of coherent structures decreases, and the energy cascade phenomenon becomes obvious. Therefore, the drag force and skin friction of a high-speed train could be reduced by increasing the velocity ratio. The dimensionless frequency *St* of the high-energy coherent structure does not change obviously and *St* = 3.09 × 10^−4^–4.51 × 10^−4^.

## Figures and Tables

**Table 1:** Experimental conditions.

Case#	*u*_c_ (m/s)	*v*_s_ (m/s)	*R*
1	30	3.9	0.13
2	40	3.2	0.08
3	40	5.2	0.13
4	40	7.2	0.18
5	50	6.5	0.13
